# Use of natural extract from HOP, thyme, orange and algae to protect potatoes against potato blight (*Phytophthora infestans*)

**DOI:** 10.1016/j.heliyon.2024.e40972

**Published:** 2024-12-05

**Authors:** Pavel Procházka, Jiří Holejšovský, Jan Řehoř, Jan Vostřel, Václav Brant, Markéta Poděbradská, Adéla Fraňková

**Affiliations:** a Department of Agroecology and Crop Production, Czech University of Life Sciences Prague, Kamycka 129, Prague 6, Czech Republic; b Department of Food Science, Czech University of Life Sciences Prague, Kamycka 129, Prague 6, Czech Republic; c Global Change Research Institute CAS - Bělidla 986/4a, Brno, 603 00, Czech Republic

**Keywords:** Potatoes, Potato blight, Natural plant protection products, Plant health, Antifungal effect, Yield quality, Pesticide substitution

## Abstract

The growing demand for alternative plant protection products (PPP) that are more environmentally friendly leads to the investigation of novel approaches in agriculture. The use of plant extracts as PPP belongs to one of the most intensively investigated areas. This study aimed to evaluate if the partial substitution of conventional pesticides with selected botanicals (seaweed extract, orange essential oil, thyme essential oil, and a hop extract) will have the same protective effect against *Phytophthora infest*ans as conventional potato treatment. The research was carried out in field conditions in two locations between 2019 and 2021. The impact of treatments on plant health status, relative chlorophyll content, and yield parameters was determined. The protective efficiency of selected natural substances (in concentrations from 0.125 to 1 %) was comparable to that of conventional fungicides. Moreover, their application reduced the use of traditional fungicides by 14–64 %, depending on the locality. In addition, the potato plants treated with the natural substances showed a higher overall and marketable tuber yield.

## 1 Introduction

Potato blight (*Phytophthora infest*ans) is one of the main diseases that cause high economic losses to potato and tomato producers due to the lower quality and yield of the crop. The annual losses in potato production are estimated to be 6 billion USD/worldwide. Depending on the epidemic situation, the disease can easily destroy 40–70 % of the potato yield [1]. The disease is caused by the pathogen *Phytophthora infestans*, which belongs to the Oomycetes group. The pathogen has different survival mechanisms [2]. During its life cycle, it can replicate through asexual reproduction by sporangia, which can directly germinate or break down into motile zoospores [3]. The primary source of the disease are tubers infected with the overwintered pathogen. After the potato planting, the mycelium grows into the above-ground plant parts. Under warm and humid conditions, sporangia are formed on the top of the plant and spread to surrounding ones by airflow. Ideal conditions for disease spread are temperature above 10 °C and relative humidity >75 % for at least two consecutive days [4,5]. Consecutively, the pathogen grows through hyphae into the plant tissues and enters the cells through haustoria used for nutrient absorption. The first symptoms of the disease occur usually a week after the infection. The dorsal part of the leaves starts showing yellow-green, later brown-black spots. Moreover, a greyish-white fungal coating formed by sporangiophores appears on the ventral part of the leaves. Later, the entire plant becomes infected and quickly dies. Tubers are again infected with sporangia washed from the infested above-ground plant parts into the soil.

Current management of the potato blight should follow an integrated pest management strategy (IPM) that relies on a combination of common-sense practices, including agrotechnical measures, use of fungicides, and cessation of vegetation. Despite that, potatoes remain one of the most fungicide dependent crops [6]. The first preventive application must be performed before the conditions become ideal for infection. After that, the application frequency of fungicide is usually every 7–10 days [7,8], depending on weather conditions and the chemicals' persistency on leaves and inside the plant.

Since the enforcement of Regulation EC 1107/2009 [9] more than 95 plant protection substances have been banned or not approved in EU [10]. Moreover, mancozeb, a broadly used fungicide in potato production, was banned in 2022 [11]. Additionally, other important pesticides are identified as candidates for substitution. Therefore, given the strong socio-political pressure to restrict certain pesticides in EU till 2030 [12], the production of the 5th most cultivated crop in EU may become highly challenging. Therefore, finding and offering farmers a wide ratio of effective and reasonable pesticide alternatives is crucial.

Currently, the use of botanical biopesticides (plant extracts) is understood as one of the strategies to reach Green Deal goals. Botanical biopesticides are compared to synthetic ones generally considered as low-risk substances for both human and environment [13,14]. Furthermore, compared with synthetic pesticides, not only can they have a direct effect on pathogens, but they can also act as plant-resistant inducers [15]. Unfortunately, their mechanism of action is due to their complexity usually unknown, which makes their registration according to current legislation [16] and consequently their marketing quite difficult. As a result, not many BPs are registered in the EU market. If so, they are usually approved for minor uses (minor crops). Due to the costly pesticide registration process, many biological substances are sold as supportive solutions (e.g., adjuvants) with vaguely specified action. Consequently, their potential for plant protection is not fully used, and farmers are often sceptical or unaware of their efficiency.

For example, algae extracts are used as biofertilizers to increase the productivity of many crops [17]. Several studies also demonstrated their activity against various plant pathogens and diseases [18,19]. However, almost any algae product on the EU market is sold as a pesticide. Moreover, various essential oils or their isolated compounds are known to be active against many plant diseases [20]. Still, the number of products containing EOs sold as pesticides is limited.

Therefore, the aim of our study was to evaluate if conventional fungicides used against potato blight can be partially substituted with selected plant extracts without a negative impact on the health and yield of potatoes. Two of the selected plant extracts are sold commercially and are based on algae and orange essential oil, the remaining ones are not commercially available (hop and thyme extract) but demonstrated promising results in the treatment of hop against *Pseudoperenospora hum*uli [21].

## 2 Results and discussion

### 2.1 The effect of treatment on the potato crop during the vegetation

**Fig. 1:**
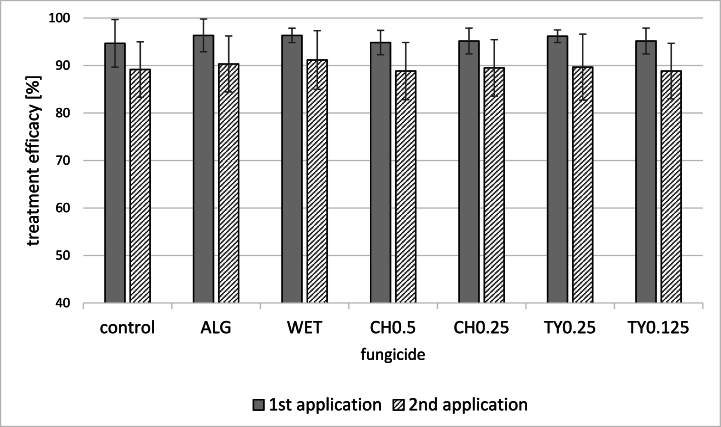
The average efficiency of fungicidal treatment (%) after the first and second fungicide application. (average of 2019–2021 and locations Chmelná, Liběšovice). No significant differences between treatments after each application were determined (p-value >0.05). AlG = Alginure (c = 1 %); WET = Wetcit (c = 0.5 %); CH0.5 = hop extract (c = 0.5 %); CH0.25 = hop extract (c = 0.25 %); TY0.25 = thyme EO (c = 0.25 %), TY0.125 = thyme EO (c = 0.125 %).

**Fig. 2:**
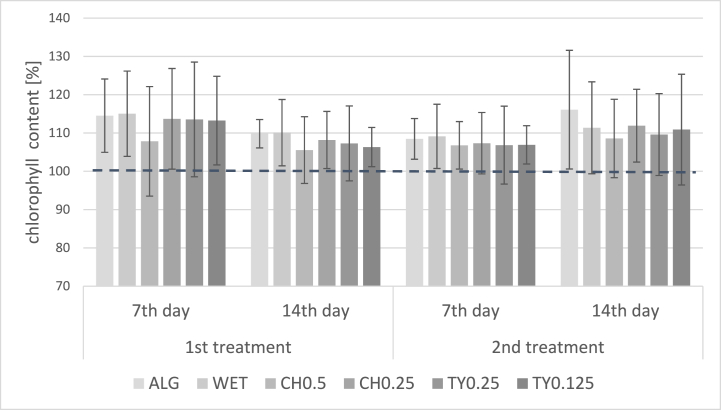
Relative chlorophyll content (%) in potato leaves after the application of experimental and conventional fungicides measured the first and second week after the application (average of 2019–2021 and locations Chmelná, Liběšovice). Significant differences between treatments were not determined (p > 0.05). AlG = Alginure (c = 1 %); WET = Wetcit (c = 0.5 %); CH0.5 = hop extract (c = 0.5 %); CH0.25 = hop extract (c = 0.25 %); TY0.25 = thyme EO (c = 0.25 %), TY0.125 = thyme EO (c = 0.125 %). The results are expressed as mean relative ratio (%) of chlorophyll content to the conventionally treated control (100 % - dash line).

In general, the partial substitution of conventional potato treatment with botanicals positively affected the observed parameters. However, the effect was not statistically significant. The percentage of leaves unaffected by *P. infest*ans infection ranged between 94 – 96 and 88–90 % after the first and second alternative treatments, respectively. The most effective were algae extract, orange, and thyme EOs (0.25 %) (Fig. 1). The efficiency of used substances after the 2nd application was lower than the first. In this case, the natural plant cell senescence could influence the results at the vegetation period's end. A similar decrease in the efficiency of the late treatments was reported for *Humulus lupu*lus [21]. All the tested substances also positively influenced the relative chlorophyll content in the leaves after both treatments. The highest increase was observed after the first treatment. The levels of chlorophyll were more than 10 % higher than the control. The only exception was the treatment with a higher concentration of hop extract (Fig. 2). The increase in pigment content can be partly explained by the fact that the application of natural substances is associated with less stress for the plant [22].

While more than 50 % inhibition of *P. infest*ans by various citrus fruits EOs (*Citrus sinensis, Citrus bergamia, Citrus li*mon with limonene, linalool, and myrcene as the main compounds) was demonstrated *in vi*tro [23], the field trials with citrus EOs brought contradictory results. El-Gamal [24] reported almost no effect of orange oil (0.75 %) against the development of the pathogen on potatoes compared to its main compounds (citral, methyl anthranate). On the other hand, commercial mixtures (i.e. Prev Am, Wetcit) containing 4–6 % orange oils were reported to be active against *P. infest*nas on various crops [25]. Moreover, those formulations can increase the crop yield by up to 50 % [26]. However, neither of those products is officially approved for potato use as a fungicide on the EU market.

Thyme EO is a wide-spectrum antimicrobial, insecticide and antifungal substance that can be highly effective against a number of economically important diseases [27]. The EO is now sold in US and Canadian markets under the name Thymox or Thyme Guard as a broad-spectrum pesticide for many crops (i.e., vegetable, fruit, hop) and a veterinary and hospital disinfectant. However, it has not been indicated for potato cultivation so far. The concentrations specified by a manufacturer for the field application range from 0.125 to 0.5 %, the higher one is more recommended. The ratio of thymol and carvacrol i.e., the main compounds of EOs in the products, is not specified. Our results demonstrated that thyme EO with thymol as a major compound can also be successfully used against the *P. infest*ans infections in doses ranging from 0.125 to 0.25 %. Interestingly, the EO also positively affected chlorophyll content in leaves (Fig. 2), although thymol is reported to affect chlorophyll synthesis in some plants negatively [28].

The efficacy of Alginure was comparable to other treatments. However, the direct effect of seaweed extract (SWE) on the pathogen remains questionable as the primary fungicidal role will probably be played by the second chemical in the preparation - potassium phosphonate (see Table 2). SWE will probably serve, thanks to their composition, as growth enhancers, activators of defense plant mechanisms, and biofertilizers. On the other hand, the antifungal activity of SWE against plant pathogens was previously reported [19].

**Table 1:** Description of main environmental characteristics of experimental locations in experimental years.

location	Year	planting date	harvest date	altitude (m a.s.l.)	average t (°C)	∑precipitation (mm)	soil type	OM (%)	pH	content of nutrients in soil∗ (ppm)			
										P	K	Mg	Ca
Chmelná∗	2019	20.4.	15.9.	485	15.1	339	cambium	2.1	5.5	55	168	75	1651
	2020	17.4.	4.10.		15.2	392		2.1	5.3	59	189	64	1548
	2021	17.4.	15.9.		14.5	419		2.1	5.5	55	170	70	1645
Liběšovice	2019	21.4.	7.9.	259	15.9	284	fluvisol	2.5	7.3	344	611	459	4400
	2020	22.4.	14.9.	266	16.2	259		2.7	7.2	282	611	459	3520
	2021	26.4.	9.9.	226	15.3	310		2.5	6.6	203	410	262	3230

Average temperature °C (IV - XI) + precipitation, OM = organic matter, content of elements in soil according to the Mehclich 3, ∗potato production area in which only specific certified and officially recognized potato varieties can be planted.

**Table 2:** Terms of botanical and conventional pesticide application in both localities.

location	year	dates of application	control treatment (CT) (dose, active ingredient)	botanicals used in experiment (dose, active ingredient)						water dose (l/ha)
Chmelná	2019	15.07.	Revus top, 0.6 (l/ha), mandipropamid, difenoconazole	Alginure, 1 %, seaweed algae extract + potassium phosphonate	Wetcit, 0.5 %, orange oil	thyme EO^a^, 0.25 %, thymol, p-cymene	thyme EO, 0.125 %, thymol, p-cymene	hop extract, 0.5 %, α-bitter acid	hop extract, 0.25 %, α-bitter acid	300
		21.08.	Infinito SC, 1.5 (l/ha), fluopikolid, propamocarb-hydrochloride							
	2020	24.06.	Revus top, 0.6 (l/ha), mandipropamid, difenoconazole							
		08.07.	Vendetta, 0.5 (l/ha), fluazim, azoxystrobin							
	2021	30.07.	Flowbrix, 2.0 (l/ha), copper oxychloride							
		18.08.	Revus top, 0.6 (l/ha), mandipropamid, difenoconazole							
Liběšovice	2019	28.06.	Flowbrix, 2.5 (l/ha), copper oxychloride							
		07.08.	Defender Dry, 2.0 (kg/ha), copper hydroxide							
	2020	17.06.	Moximate 725 WG, 2.5 (kg/ha), mancozeb, cymoxanil							
		14.08.	Defender Dry, 2.0 (l/ha), copper hydroxide							
	2021	27.07.	Flowbrix, 2.0 (l/ha), copper oxychloride							
		20.08.	Revus top, 0.6 (l/ha), mandipropamid, difenoconazole							

a EO – essential oil.

So far, the use of hop extract as a plant protectant has not been widely investigated. However, interest in the antimicrobial activity of its biologically active compounds is increasing. The anti-feedant activity of hops against various pests has been reported and attributed to primary prenylated flavonoid content [29]. Also, its antifungal activity against *Zymoseptoria trit*ici, causing Septoria tritici blotch, has been demonstrated [30]. Another research confirmed its potential in hop protection against *Pseudoperenospora humuli* [21]. Quite recently, *in vitro* activity of hop extract and isolated bitter acids prepared from different plant parts against various growth stages of *P. infestans* was confirmed [31]. The results from our field trials confirm that the hop extract (0.25 %) can be successfully used to protect potatoes.

The mechanism of action of tested plant extracts is yet to be discovered. From the previous studies, it can be assumed that all of them can act as plant strengtheners and fungicides. Regarding the EOs, the direct fungicidal effect will probably dominate, while SWE will act more as a plant strengthener. Several compounds from SWE have already been identified to act as positive plant growth regulators or metabolic enhancers [32]. Stirk and van Staden [33] have also demonstrated increased chlorophyll content in plants after applying *Ascophyllum nodosum* L. extract in tomato, wheat, and maize plants.

Interestingly, the use of alternative treatments led, on average, to a 14–64 % reduction in the application of synthetic fungicides (see Appendix 1) in Chemlná and Liběšovice, respectively. The high fluctuation is given by the differences in the infectious pressure, locality, and the length of the cultivation period.

### 2.2 The effect of treatment on the yield and potato size

**Fig. 3:**
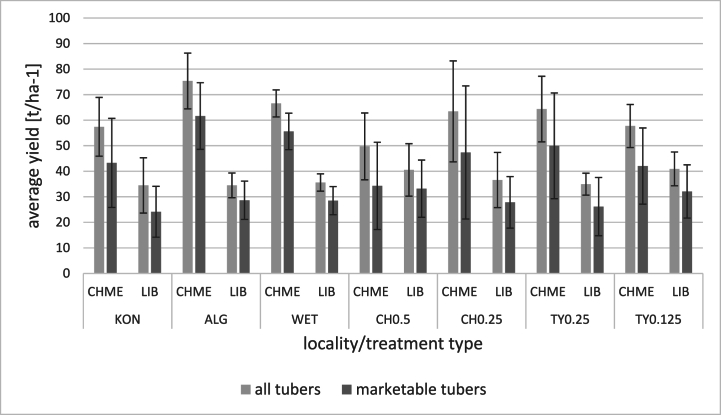
Average yield of marketable and all potato tubers (t.ha^−1^) treated by conventional and experimental pesticides (average of 2019–2021 for Chmelná and Liběššovice). Significant differences between treatments were not determined (p > 0.05). Results are expressed as mean ± standard deviation. KON = control variant, AlG = Alginure (c = 1 %); WET = Wetcit (c = 0.5 %); CH0.5 = hop extract (c = 0.5 %); CH0.25 = hop extract (c = 0.25 %); TY0.25 = thyme EO (c = 0.25 %), TY0.125 = thyme EO (c = 0.125 %). CHME – locality Chmelná, LIB – locality Liběšovice.

**Fig. 4:**
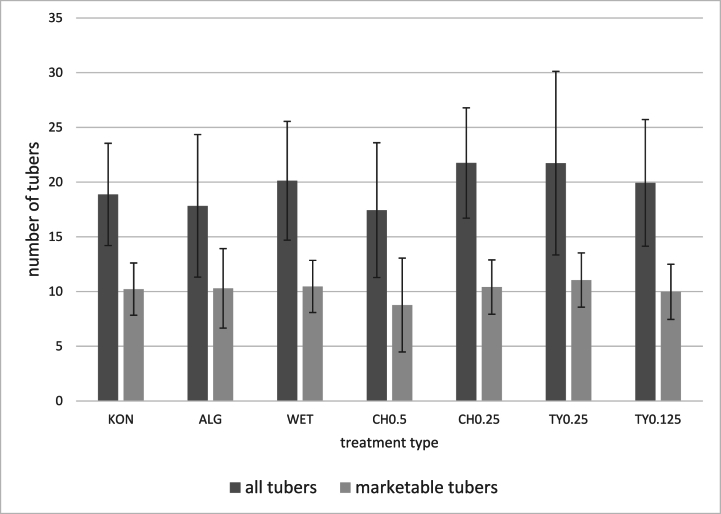
Average number of all and marketable tubers under one potato plant (average of 2019–2021 for locations Chmelná and Liběšovice). Significant differences between treatments were not determined (p > 0.05). The results are expressed as mean ± standard deviation. KON = control variant, AlG = Alginure (c = 1 %); WET = Wetcit (c = 0.5 %); CH0.5 = hop extract (c = 0.5 %); CH0.25 = hop extract (c = 0.25 %); TY0.25 = thyme EO (c = 0.25 %), TY0.125 = thyme EO (c = 0.125 %).

In general, treating potatoes with natural substances did not statistically affect the overall yield and yield of marketable tubers. The average yield differed by the locality and was approx. 1.5 times higher at Chmelná (47 t ha^−1^), which is a more suitable location for potato cultivation than Liběšovice. Plants treated with natural substances had higher total and marketable tuber yields than control. The only exception were potatoes treated with 0.5 % hop extract at Chmelná (Fig. 3). Moreover, the number of tubers under one plant remained unaffected (Fig. 4). The potatoes treated with Alginure had the highest average yield (average of three years = 75 t ha^−1^) followed by Wetcit, hop extract, and thyme oil applied in their lower concentrations. The positive effect of algae and thymol, carvone, and eugenol on potato yield compared to conventional fungicides was previously demonstrated [27,34]. The effect of orange oil on the potato yield has not been reported before.

## 3 Conclusions

The field trials demonstrated that partial substitution of conventional fungicides with natural substances exhibited comparable effectiveness against the development of potato blight vegetation that of the conventional treatment. Moreover, plants treated with natural substances had higher relative chlorophyll content and higher total and marketable tuber yield than control. The average number of potato tubers under one plan was not affected. The most effective natural substances were seaweed extract and orange oil, followed by lower concentrations of thyme EO (0.125 %) and hop (0.25 %) extract. Last but not least, two alternative treatment applications reduced fungicides' use from 14 % up to 64 %, depending on the locality and environmental conditions. Therefore, the selected natural substances can decrease the environmental burden caused by potato production and should be considered by potato producers as an adequate partial substitution for conventional fungicides.

## 4 Materials and methods

The three-year experiment was run from 2019 to 2021. During the potato vegetative period, two doses of conventional fungicides were every year substituted with different concentrations of 4 botanicals (hop extract, thyme oil, orange oil and algae extract). Their ability to protect potatoes from *P. infestans* was evaluated and compared with conventional treatment. Moreover, their influence on the yield quality was determined.

### 4.1 Experimental locations

The experiments took place at two locations in the Czech Republic: Chmelná (49.6481022N, 14.9883519E) and Liběšovice (50.2452906N, 13.5005919E). These locations were selected to demonstrate the effectiveness of alternative treatments under various environmental conditions and infection pressure. Chmelná is in a potato growing area with higher humidity and generally high infectious pressure of *P. infestans* compared to the second location. Detailed area description is given in Table 1.

### 4.2 Plant material and layout of the field trials

Two potato varieties commonly cultivated in the Czech Republic, ‘Adela’ and ‘Antonia’, were grown at the Chmelná and Liběšovice locations, respectively. These varieties exhibit relatively high resistance to fungal pathogens, with ‘Adela’ rated 8/9 and ‘Antonia’ 7/9. This characteristic aligns with one of the fundamental requirements for cultivation under integrated pest management (IPM) principles. However, around 5–10 fungicidal treatments are still required per their cultivation cycle. The varieties were selected based on their agroecological suitability for the respective locations. ‘Adela’ is an early-season potato of cooking type B, a versatile tuber suitable for cooking, baking, and frying. It has deep yellow flesh, yellow-brown skin, and oval-shaped tubers. In contrast, ‘Antonia’ is a semi-early variety of cooking type A, ideal for salads and side dishes, with yellow flesh, yellow skin, and oval tubers.

The field trials were organized in a randomized block design with three replications. Each block consisted of seven experimental plots, each measuring 2.25 m in width by 10 m in length. The width of each plot corresponded to three rows of potato plants, with a 130 cm space between plots, equivalent to the width of two potato rows. Planting dates for the tubers (see Table 2) were adjusted based on prevailing weather conditions. Land preparation was tailored to the specific requirements of each location. A detailed description of the agrotechnical practices and the application of protective substances before and during the growing season at the experimental sites is provided in Appendices 1A and 1B.

### 4.3 Experimental treatment of potatoes

Six experimental and one control application sequences of pesticides were tested during the potato vegetative period. The application sequence consisted of 5–10 fungicide applications depending on the year, location, and infectious pressure (Appendix 1A and B). Experimental variants, 4 botanicals (in concentrations from 0.125 to 1 %) altered two inputs of a conventional fungicide. The orange oil (Wetcit - orange oil 4.2 %,OroAgri, NL) and algae extract (Alginure - seaweed algae extract 24 % + 26.3 % potassium phosphonate), Biocont, CZ) are commercially available. Therefore, they were applied according to the manufacturer's specifications. Hop extract and thyme oil formulations were developed in-house and are unavailable on the market. The base for the hop formulation was CO_2_ hop extract (Hopsteiner, DE) standardized by producer to 50 % of α-bitter acid. For the preparation of the thyme formulation, thyme essential (Sigma Aldrich, CZ) oil with the thymol (44 %) and p-cymene (29 %) as the main identified compounds was used. The fungicidal application followed the methodology of The Czech Potato Research Institute [35], which reflects agroecological condition of the Czech Republic. According to the methodology, the fungicidal applications are divided to preventive (i.e. before the incidence of the disease) and curative (when the symptoms of diesease are visible). The first application of fungicide is recommended before the spores from primarily infected plants are spread, this corresponds to the stage when the plants start to meet between the rows. Consecutive fungicidal treatment should be applied in 7–10 (14) day depending on the ‘index of prognosis and signalization of the infection’. To evaluate curative effect of natural substances, they were firstly applied when the first symptoms of the disease were visible (BBCH 66–69). The second application followed after two to three treatments with conventional fungicides (BBCH 75–79). This pesticide rotation should prevent from the developing of the resistance to tested compounds.An overview of all variants together with applied concentrations are provided in Table 2.

### 4.4 Determination of treatment efficiency

The protection efficiency of alternative treatments against *P. infestans* followed the European and Mediterranean Plant Protection Organization standards [36]. Firstly, the ratio of infected to healthy plants was evaluated on a scale of 0–100 % on 100 randomly selected plants in each replication a week after substance application. The healthy plant was defined as plant without visible symptoms of disease on stems and leaves. The bio-stimulatory effect of the pesticides was assessed by determining chlorophyll content one and two weeks after each alternative pesticide application. Yara N tester (UK) was used for this purpose. The average values were calculated from 30 measurements for each treatment. The values were subsequently converted to the relative chlorophyll content. The values obtained for control treatment, i.e., the conventional fungicidal protection, were used as reference.

The potatoes were always yielded from the entire plot in full maturity when all the stems and leaves were dead (the BBCH 97–99). The number of the plants in each plot was determined before harvest. The influence of the treatments on the yield quality was evaluated by three parameters: 1) Yield of potato tubers (t/ha) extrapoletd from the yield of entire plot; 2) yield of marketable tubers (size >28 mm according); 3) an average number of all tubers and marketable tubers under each plant.

### 4.5 Statistical evaluation

Statistical analyses were carried out in Statgraphics®Plus 4.0 (Warrenton, USA). Analysis of variance (ANOVA, Tukey test, p-value) was used to evaluate the efficiency of various treatments.

## CRediT authorship contribution statement

**Pavel Procházka:** Writing – review & editing, Supervision, Investigation, Formal analysis, Data curation, Conceptualization. **Jiří Holejšovský:** Investigation. **Jan Řehoř:** Writing – original draft, Investigation. **Jan Vostřel:** Writing – original draft, Investigation. **Václav Brant:** Supervision, Data curation. **Markéta Poděbradská:** Writing – review & editing. **Adéla Fraňková:** Writing – review & editing, Supervision, Funding acquisition, Formal analysis, Data curation, Conceptualization.

## Data availability statement

Data will be made available on request.

## Declaration of competing interest

The authors declare that they have no known competing financial interests or personal relationships that could have appeared to influence the work reported in this paper.

## Figures and Tables

**Table:** 

operation/application	2019		2020		2021	
disking + catch crops sowing		July 30, 2018		August 01, 2019		August 03, 2020
catch crops mulching		October 29, 2018		October 30, 2019		October 27, 2020
application of manure + ploughing to 28 cm		November 30, 2018; dose 38 t/ha		October 30, 2019; dose 35 t/ha		October 28, 2020; dose 35 t/ha
grooving rough furrows + separation soil clods and stones		April 19, 2019		April 16, 2020		April 16, 2021
planting		April 20, 2019		April 17, 2020		April 17, 2021
herbicide treatment	23.04.	Bandur (aclonifen); 4 L/ha	7.5.	Arcade 880 EC (prosulfocarb, metribuzin); 5 L/ha	29.4.	Arcade 880 EC (prosulfocarb, metribuzin); 5 L/ha
1. application	02.06.	I: fluopicolide: Proteus 110 OD (thiacloprid, deltamethrin); 0.5 L/ha FT: Galleko univesal; 0.5 L/ha	13.6.	F: Ridomil Gold MZ Pepite (mancozeb, metalaxyl-M); 2.5 kg/ha FT: MgS; 10 kg/ha + Urea; 10 kg/ha + K-fenol mix; 0.2 L/ha		F: Ridomil Gold MZ Pepite (mancozeb, metalaxyl-M); 2.5 kg/ha FT: MgS; 10 kg/ha + Urea; 10 kg/ha
2. application	18.06.	F: Ridomil Gold MZ Pepite (mancozeb, metalaxyl); 2.5 kg/ha	24.6.	F: **experimental variants** (see Table 2) and Revus Top (mandipropamid, difenoconazole); 0.6 L/ha FT: Kombiphos; 2 L/ha + K-gel; 175 1.5 L/ha + K-fenol mix 0.2; L/ha + MgS; 10 kg/ha	22.6.	F: Revus Top (mandipropamid, difenoconazole); 0.6 L/ha FT: Kombiphos; 2 L/ha + K-gel 175; 1.5 L/ha + K-fenol mix; 0.2 L/ha + MgS; 10 kg/ha
3. application	30.6	F: Ridomil Gold MZ Pepite (mancozeb, metalaxyl); 2.5 kg/ha FT: Galleko universal; 0.5 L/ha + K-gel 175; 3.5 L/ha Urea; 5 kg/ha	1.7.	F: Ridomil Gold MZ Pepite (mancozeb, metalaxyl-M); 2.5 kg/ha FT: MgS; 10 kg/ha + Urea; 10 kg/ha + K-gel; 175; 1.5 L/ha	30.6.	F: Revus Top (mandipropamid, difenoconazole); 0.6 L/ha FT: K-gel 175 1.5 L/ha + K-fenol mix; 0.2 L/ha + MgS; 10 kg/ha
4. application	15.7.	F: **experimental variant**s (see Table 2) and Revus top (difenoconazole, mandipropamid); 0.6 L/ha I: Vaztak activ (alfa-cypermethrin); 0.25 L/ha	8.7.	F: **experimental variants** (see Table 2) and Vendetta (fluazinam, azoxystrobin); 0.5 L/ha I: Ecail Ultra (thiacloprid); 0.2 L/ha FT: MgS 10 kg/ha + Kombiphos; 2.5 L/ha	7.7.	F: Vendetta (fluazinam, azoxystrobin); 0.5 L/ha I: Ecail Ultra (thiacloprid); 0.2 L/ha FT: 10 MgS kg/ha + Kombiphos 2.5 L/ha
5. application	29.07.	F: Infinito SC (fluopicolide, propamocarb-hydrochlorid); 1.2 L/ha I: Ecail Ultra (thiacloprid); 0.2 L/ha FT: Urea; 5 kg/ha	14.7.	F: Acrobat MZ WG (mancozeb, dimethomorph); 2 kg/ha FT: Galleko leaf; 0.4 L/ha + K- fenol mix; 0.2 L/ha + Kombiphos; 2 L/ha	14.7.	F: Infinito (fluopicolide, propamocarb-hydrochloride); 1.5 L/ha FT: Galleko leaf; 0.4 L/ha + MgS; 10 kg/ha
6. application	11.8.	F: Altima 500 SC (fluazinam); dose 0.3 L/ha FT: K-gel 175; 3.5 L/ha	20.7.	F: Infinito (fluopicolide, propamocarb-hydrochloride); 1.5 L/ha FT: Galleko leaf; 0.4 L/ha + MgS; 10 kg/ha + K-gel 175; 3 L/ha	20.7.	F: Acrobat MZ WG (mancozeb, dimethomorph); 2 kg/ha FT: Galleko leaf; 0.4 L/ha + K- fenol mix; 0.2 L/ha + Kombiphos; 2 L/ha
7. application	21.8.	F: **experimental variants** (see Table 2) and Infinito SC (fluopicolide, propamocarb-hydrochloride); 1.5 L/ha FT: K-gel 175, dose 3.5 L/ha	27.7.	F: Revus Top (mandipropamid, difenoconazole); 0.6 L/ha FT: Galleko leaf; 0.4 L/ha + K-fenol mix; 0.2 L/ha + Kombiphos; 2 L/ha	30.7.	F: **experimental variants** (see Table 2) and Revus Top (mandipropamid, difenoconazole); 0.6 L/ha FT: Galleko leaf; 0.4 L/ha + K-fenol mix; 0.2 L/ha + Kombiphos; 2 L/ha
8. application	28.8.	F: Altima 500 SC (fluazinam); 0.3 L/ha	4.8.	F: Altima 500 SC (fluazinam); 0.4 L/ha FT: MgS; 10 kg/ha + K-gel 175; 3 L/ha	4.8.	F: Altima 500 SC (fluazinam); 0.4 L/ha FT: MgS; 10 kg/ha + K-gel 175; 3 L/ha
9. application		not used	10.8.	F: **experimental variants** (see Table 2) and Infinito SC (fluopicolide, propamocarb-hydrochloride); 1.5 L/ha FT: K- gel 175; 2 L/ha	11.8.	F: Infinito (fluopicolide, propamocarb-hydrochloride); 1.5 L/ha FT: K- gel 175; 2 L/ha
10. application		not used	19.8.	F: Altima 500 SC (fluazinam); 0.3 L/ha	19.8.	F: **experimental variants** (see Table 2) and Altima 500 SC (fluazinam); 0.3 L/ha
harvest + yield evaluation		September 15, 2019		October 04, 2020		September 19, 2021

F – fungicide; FT: fertilizer; I – insecticide.

**Table:** 

operation/application	2019		2020		2021	
disking		July 28, 2018		August 06, 2019		August 15, 2020
application of compost + ploughing to 25 cm		November 30, 2018; dose 20 t/ha		October 24, 2019; dose 20 t/ha		October 27, 2020; dose 20 t/ha
2 times combined cultivator to 15 cm		April 19, 2019		April 21, 2020		April 24, 2021
planting		April 21, 2019		April 22, 2020		April 26, 2021
application of herbicide	07.05.	Citation (metribuzin); 0.5 kg/ha	09.05.	Citation (metribuzin); 0.5 kg/ha	07.05.	Arcade 880 EC (prosulfocarb, metribuzin); 5 L/ha
application of fertillizer	12.05.	Plant Activ; 1.0 kg/ha + Amoniium nitrate with urea; 40 kg/ha	12.05.	Plant Activ; 1.0 kg/ha + Amoniium nitrate with urea; 60 kg/ha	14.05.	Plant Activ; 1.0 kg/ha + Amoniium nitrate with urea; 260 kg/ha
1. application	28.06.	F: **experimental variants** (see Table 2) and Flowbrix (oxychlorid Cu); 2.5 kg/ha LF: Yara Calcinit; 12.5 L/ha + Yara Zintrac; 1.0 L/ha	17.06.	F: **experimental variants** (see Table 2) and Moximate 725 WG (mancozeb, cymoxanile); 2.5 kg/ha LF: Yara Calcinit; 12.5 L/ha + Yara Zintrac; 1.0 L/ha	20.06.	F: Revus top (difenoconazole, mandipropamid); 0.6 L/ha
2. application	03.07.	I: Coragen 20 SC (chlorantraniliprole); 0.05 L/ha	03.07.	I: Coragen 20 SC (chlorantraniliprole); 0.05 L/ha	24.06.	I: Vaztak activ (alfa-cypermethrin); 0.25 L/ha
3. application	13.07.	F: Revus top (difenoconazole, mandipropamid); 0.6 L/ha I: Vaztak activ (alfa-cypermethrin); 0.25 L/ha	15.07.	F: Revus top (difenoconazole, mandipropamid); 0.6 L/ha + Kuprikol 50 (oxychloride Cu); 2.0 L/ha FT: Urea; 10.0 kg/ha + Yara Thiotrac; 4 L/ha	14.07.	F: **experimental variants** (see Table 2) and Flowbrix (oxychloride Cu); 2.0 L/ha
4. application	07.08.	F: **experimental variants** (Table 2) and Defender Dry (hydroxyde Cu); 2.0 kg/ha	14.08.	F: **experimental variants** (see Table 2) and Defender Dry (hydroxyde Cu); 2.0 kg/ha	27.07.	F: Acrobat MZ WG (mancozeb, dimethomorph); 2 kg/ha FT: Galleko leaf 0.4 L/ha + K- fenol mix 0.2 L/ha + Kombiphos; 2 L/ha
5. application		not used		not used	20.08.	F: **experimental variants** (see Table 2) and Revus Top (mandipropamid, difenoconazole); 0.6 L/ha
harvest + yield evaluation		September 07, 2019		September 14, 2020		September 09, 2021

F – fungicide; FT: fertilizer; I – insecticide.

## Appendix 1A

Overview of the field operation during the years 2019–2021 at Chmelná.
